# Beyond the Food Systems Summit: Linking Recommendations to Action—The True Cost of Food^☆^

**DOI:** 10.1016/j.cdnut.2023.100028

**Published:** 2023-02-08

**Authors:** Eileen T. Kennedy, Maximo A. Torero, Dariush Mozaffarian, William A. Masters, Roy A. Steiner, Sheryl L. Hendriks, Jamie A. Morrison, Kathleen K. Merrigan, Shibani A. Ghosh, Daniel E. Mason-d’Croz

**Affiliations:** 1 Friedman School of Nutrition Science and Policy, Tufts University, Boston, MA, United States; 2 Food and Agriculture Organization, Rome, Italy; 3 Rockefeller Foundation, New York, NY, United States; 4 Department of Agricultural Economics, Extension and Rural Development, University of Pretoria, Lynnwood Road, Hatfield, South Africa; 5 Global Alliance for Improved Nutrition, Geneva, Switzerland; 6 Swette Center for Sustainable Food Systems, Arizona State University, Tempe, AZ, United States; 7 College of Agriculture and Life Sciences, Cornell University, Ithaca, NY, United States

**Keywords:** true cost accounting, true value of food, United Nations Food Systems Summit, metrics, agriculture, nutrition

## Abstract

A transformation of food systems is needed to achieve the 17 Sustainable Development Goals specified in the 2030 Agenda for Sustainable Development. Recognizing the true costs and benefits of food production and consumption can help guide public policy decisions to effectively transform food systems in support of sustainable healthy diets. A new, expanded framework is presented that allows the quantification of costs and benefits in three domains: health, environmental, and social. The implications for policy makers are discussed. *Curr Dev Nutr* 2023;x:xx.

## Introduction: True Cost Accounting and the True Cost of Food

The need for true cost accounting (TCA) in food systems was highlighted in the concluding Statement of Action from the United Nations Food Systems Summit (UNFSS), which noted that “the value of food must be realized, and the economic, social, and environmental impact and externalities must be better assessed and mitigated or leveraged as required” [1]. This statement was reinforced by a series of articles and events preceding the Summit including the Scientific Group’s June 2021 article on True Cost and True Price of Food [2] and the Rockefeller Foundation’s assessment of the true cost of the United States food system [3]. Building on these significant reports, the UNFSS has provided the momentum to advance knowledge that translates theory into practice related to TCA, a method that accounts for all costs and benefits of a product, including externalities that harm or help other people through the environmental, social, and health consequences of production and consumption [3].

Numerous unintended consequences of modern food systems are externalities not reflected in prices paid by consumers or received by producers, creating gaps between current market incentives and the true cost of food. Many external harms are illustrated vividly in the 2021 Rockefeller Foundation Report [3]: the United States spends $1.1 trillion per year directly on food, but the effect of poor diet quality on health care costs in addition to the negative effects of harmful production systems on the environment and loss of biodiversity is estimated to cost an additional $2.2 trillion or ∼$2 in negative externalities for every $1 spent on food. The Scientific Group [2] of the USFSS made similar observations for the world food system, noting that market prices paid by consumers for harmful products would be significantly higher if the cost of negative externalities were included, whereas healthy foods would have higher (this should be lower market prices) market prices for consumers, and regenerative agricultural practices would have higher returns to producers. Because different foods and food system strategies can create positive or negative economic effect, and every choice has tradeoffs and likely unintended consequences for the environment, health, livelihoods, and society, there is an urgent imperative to better quantify the entire range of costs and benefits to inform priorities for the successful transformation of food systems.

To be sustainable, food system transformation requires achieving a system that is good for people (for example, for health and well-being), the environment (for example, for climate, water use, soil, land, oceans, and biodiversity), and the society (that is, for culture, equity, and justice). TCA forms the basis for estimating such costs. The approach can identify the critical “hidden costs” or the negative externalities that inflict costs on health, the environment, and society. Importantly, a TCA approach can similarly identify the critical “hidden benefits” or positive externalities that create good for the world. Then, these costs delete second "then" from textthen need to be modeled to determine the likely effects of any change in the system (for better or worse). Thus, a distinctive feature of TCA is addressing both the true costs and the true benefits of food. When both negative and positive externalities (value) are captured, the true value of many foods may be lower than their market price, even as other foods have true costs above their market price. This is foundational to understand because, as the OECD [4] has defined, externalities are “situations when the effect of production or consumption of goods and services imposes costs or benefits on others which are not reflected in the prices charged for the goods and services provided.” The degree to which some aspects of modern food systems contribute to poor health, climate change, deforestation, population disparities, and cause other harms, whereas beneficial activities contribute to improvements, is not factored into standard indicators used by decision makers. This gap between market prices and societal needs promotes a value-destroying food system, creating the need for new metrics to guide value creation.

TCA provides a useful tool for governments and the private sector to meet the 17 Sustainable Development Goals [5]. Although still in its early stages, TCA has enormous potential to help decision makers set priorities and implement actions to maximize the positive health, environmental, and societal benefits of food and nutrition policies and programs and minimize unintended harms to health, environment, and society. Because transforming the food system to meet health, environmental, and social goals is a recognized priority for many governments, one outcome of the UNFSS must be wider recognition and adoption of appreciating the true value of food to guide system changes. To effectively accomplish this requires, an in-depth understanding of the types of data needed, methodological approaches, and applications within a specific national or subnational context.

One of the biggest challenges for TCA in the food system is to trace the many diverse implications of food production, distribution, and consumption for the environment, health, and society. Advances in climate modeling and studies of natural resource depletion from harmful agricultural practices, combined with epidemiologic studies of elevated disease risks from poor diet quality, have clearly identified a variety of negative environmental and health externalities, whereas other external costs or benefits are less well measured. The effective application of true value of food systems approaches will necessitate development and testing of novel sets of metrics that will capture the complexity of calculating the true value of food. Thus, in addition to the importance of using true value–based approaches on current evidence, there must be significant government and private sector investment in further fundamental and applied research to identify and quantify aspects of externalities and, where needed, to provide new mechanisms for generating data for rigorous analysis. A TCA approach is discussed further.

## Cost and Affordability of Healthy Diets: Linking Economics to Nutrition and Well-being

**FIGURE 1:**
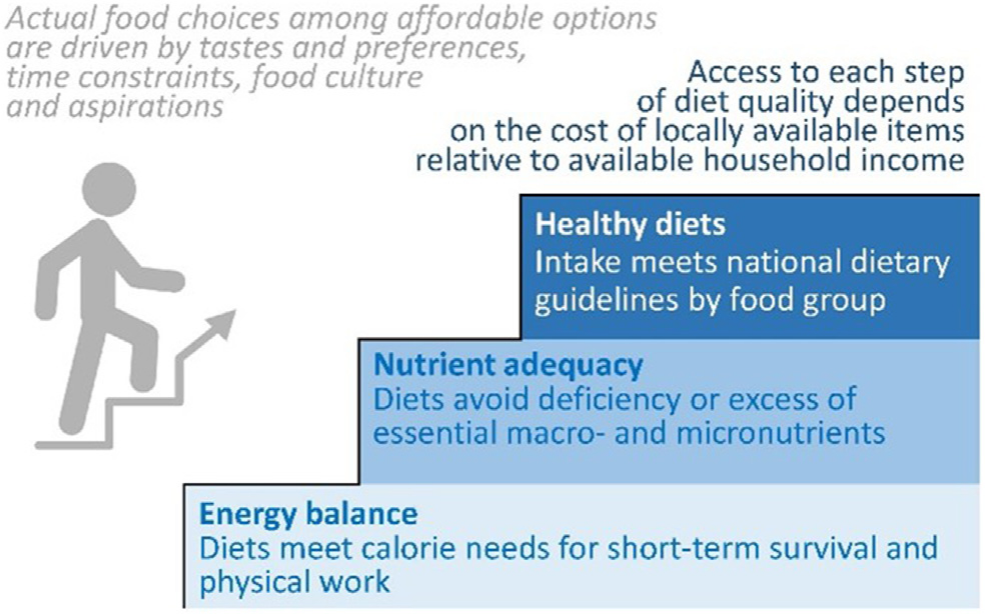
The staircase approach to measuring access as affordability of least-cost diets. Note: The use of least-cost items by food group as a metric of access to healthy diets across countries and over time was initiated by Masters et al. [6], with this staircase model of access to each level of diet quality first published in Herforth et al. [7] by the Food Prices for Nutrition project (https://sites.tufts.edu/foodpricesfornutrition).

Ensuring healthy diets, particularly for low-income populations, requires production, access, affordability, and convenience. Figure 1 [6,7] displays the various ways that healthy diets have been defined, using the staircase model developed by the Food Prices for Nutrition project to illustrate differences in the market cost of retail food items needed to reach each standard of diet quality [8]. The first step is to maintain energy balance for work each day, establishing the baseline cost of caloric adequacy to prevent hunger and starvation. The second step up is a balanced diet of essential nutrients, with enough diversification to avoid deficiencies and excesses of the roughly 2 dozen macronutrients and micronutrients for which upper and lower bounds are known. A third step up is balanced food groups, bringing other attributes of a healthy diet specified in national food-based dietary guidelines. Measuring physical and economic access to each step of the ladder is measured using the least expensive items to meet that standard, identifying variation in which foods provide the least-cost way to meet nutritional requirements as pioneered by work in Ghana and Tanzania [6]. Comparing least-cost diets per day to household income available for food reveals whether the dietary patterns we actually observed are caused by lack of physical and economic access to healthy items or by food choices from among the affordable options.

As shown in Figure 1, the overarching choice is driven by factors other than nutrients or health such as taste and preferences, time use and meal preparation practices, food culture, and aspirations. The core finding of Food Prices for Nutrition is that, on average across the globe using the most recent complete price data, least-cost items for each step of the ladder cost an average of $0.83, $2.46, and $3.75 per day respectively, whereas actual food expenditure varies widely; observed spending on food is often less than half what would be needed for a least-cost healthy diet in the world’s poorest countries and more than three times the minimum cost of a healthy diet in many high-income countries where actual food choices are often both more expensive and less healthy than the least-cost ways of following national dietary guidelines [8]. Overall, ∼3 billion people or roughly 38% of the world’s population cannot afford a healthy diet [9]. Most of these people live in sub-Saharan Africa, South, and South-East Asia, whereas people in high-income settings who often spend >$10 per day on food may buy items that have both lower nutritional value and higher environmental costs than would be needed for a healthy diet. There is often the perception that consuming a healthy and sustainable diet is more expensive than what people currently consume, but actually, calculating least-cost healthy diets reveals that the least expensive items have low market prices primarily because they use relatively little energy, farmland, water, or other resources, thus limiting their carbon footprint and other negative externalities compared with actual consumption levels of high-cost foods such as beef that are consumed for reasons other than health [7]. Diet costs need not be more expensive to be more sustainable, but the cost of nutrient adequacy does vary by demographic group because costs per day are highest for adolescents and women and cost per calorie are greatest for girls and women owing to their higher nutrient needs [10].

**FIGURE 2:**
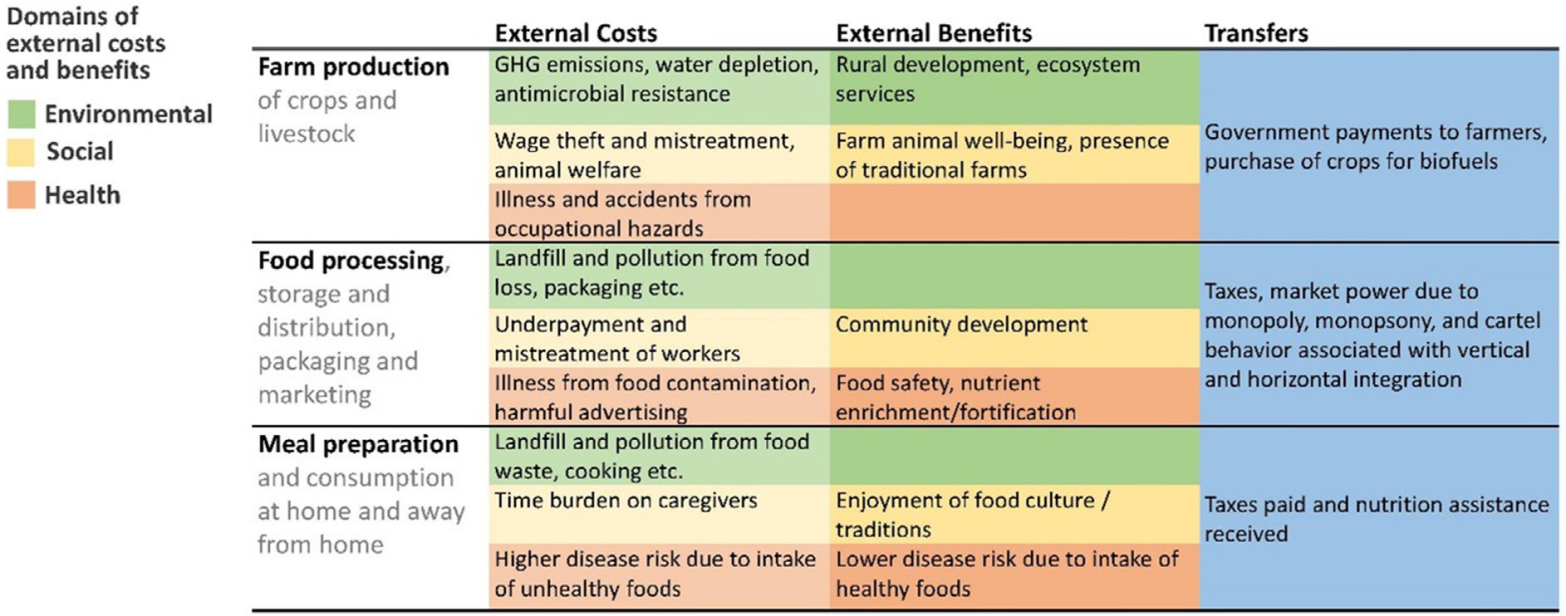
Structure and example elements for true cost accounting to guide food choices. Note: Rows are grouped by stage of product life cycle, from farm production through postharvest transformation and delivery, to final use in meal preparation and consumption. Colors indicate the three domains of external costs, defined in terms of environmental sustainability and natural resource use in green, social sustainability and equity in yellow, and health outcomes for the population in orange. Columns show examples of external costs, external benefits, and transfers within the population where one person’s loss is another person’s gain. This accounting framework was developed for Martinez and Masters [11] as part of the Food Prices for Nutrition project (https://sites.tufts.edu/foodpricesfornutrition). GHG, greenhouse gas.

Beyond the data and methods needed for measuring access to least-cost healthy diets, as noted by Martinez and Masters [11], “To build sustainable, inclusive and resilient food systems that provide, healthy affordable food for all people at all times, governments will need expanded accounting frameworks that consider gains from beneficial practices, in addition to the costs of harmful activities.” TCA is increasingly being adopted to evaluate food-related choices complementing market prices with data on nonmarket effects of food system activities. To that end, an expanded framework is proposed in Figure 2 that addresses the effects of food systems on nutrition and health at each stage of the food supply from farm to household consumption. The main domains affecting these external costs and benefits are health, environmental, and social—with both costs and benefits elucidated to accurately measure the true cost of food. Equally important is the inclusion of potential government actions that can minimize costs and enhance benefits. One example from Ethiopia is a program providing seeds and technical assistance in changing the crops produced to include cultivation of nutrient-rich fruits and vegetables [12]. The program was intended to decrease the environmental insults from the overcultivation of animal source foods and vary the commodities produced while improving diet quality through consumption of more nutrient-rich foods. Other analyses of government food and nutrition actions that incorporate economic costs on health have been reported for both undernutrition and chronic diseases, across diverse nations such as India, Nigeria, Ethiopia, Argentina, and the United States [[13], [14], [15]]. The proposed framework thereby expands significantly on existing efforts at TCA, such as Minotti et al. [16] for Italy.

## From Theory to Practice: A TCA Approach to Policies and Programs

TCA is a tool that could be highly informative and effective for national government decision making in the food sector, building on the longstanding use of cost-benefit analysis (CBA) in other sectors. CBA aims to provide objectivity in assessing implications of proposed projects, programs, and policies, based on the concept that benefits should outweigh costs and that decision makers should prioritize activities with greater benefits relative to their costs [12]. In extending CBA to TCA in the food sector, three challenges must be considered and addressed.

The first obstacle to overcome is the nature and extent of available data. CBA focuses on the difference between benefits and costs for a specific action relative to some baseline alternative. If TCA is to guide action in the food sector, costs and benefits must be similarly explicit about the alternatives being considered, so as to identify the more beneficial or less costly options. Because TCA for food is in its infancy, there are data gaps that, at the moment, preclude more widespread application for certain interventions, externalities, or national contexts. One example is the 2021 Rockefeller Foundation report [3] where costs of inequity, although known to be important, were not included in the 14 variables used for TCA because of inability to quantify this externality. There are other nontraded, nonmarket benefits that cannot always be reliably quantified, such as biodiversity.

A second challenge is the analytical complexity of using TCA as part of decision making. In the activities leading up to the UNFSS, including the series of independent dialogs and the Summit itself, there was a consistent call for more appropriate metrics to effectively monitor and evaluate the process and effect of transforming food systems. The complex information needs included the proper types of data, utility of various indicators, relationships across measures, changes over time, and the levels of precision and accuracy to ensure reliability, completeness, weighting, and interpretation. This articulated list of needs could be seen as overwhelming. These analyses cannot be static. As new and improved assessments of the core elements contained in the analysis are updated, so these assessments need to be updated with revised data estimates. However, the importance of TCA calls for coordinated efforts and investments to begin to collate these data.

A final set of challenges involve implementation. As a formal decision-making tool, TCA can require in-depth analysis of information, which requires time and resources, which governments have often not currently set aside. In addition, the quantification of complex, multidimensional data requires diverse skill sets that may need collaboration and coordination across diverse sectors and institutions.

In summary, we believe that the power of TCA described warrants addressing these challenges and concerns in a systematic manner. Qualitative judgment is needed, together with a broad conceptual framework to ensure that a wide range of factors is considered when guiding policies and programs toward a more healthy, sustainable, and inclusive food systems. The incorporation of externalities into the true price of food must include the benefits for healthy, sustainable foods, helping to reduce their price and address food and nutrition insecurity in already vulnerable global populations.

## Conclusion

A true value approach to the costs and benefits of policy choices provides a powerful tool for the effective transformation of food systems. Its use requires multidisciplinary expertise in nutrition, environmental science, public health, economics, epidemiology, agriculture, statistics, data management, and more. Building on the UNFSS and SDG, we believe it is time for a unified framework for applying TCA to food systems. A part of this process will be the identification and development of newer data sources, methods, and metrics. The methods that are ultimately recommended should allow decision makers to see benefits and costs associated with each choice, with increasing degrees of precision and usefulness as the field advances. Simultaneously, the need for a comprehensive approach should not discourage incremental steps that build on and improve the existing methods. The UNFSS and presummit activities created momentum for ensuring that food systems provide healthy, sustainable, inclusive diets. TCA can help leverage and catalyze this momentum for meaningful, evidence-based food system transformation.

## Funding

No external sources of funding supported the development of this study, which summarizes results of a webinar discussion among the authors hosted at Tufts University on April 25, 2022.

## Author disclosures

None of the authors have a conflict of interest to report.
